# Mental fatigue assessment may add information after aneurysmal subarachnoid hemorrhage

**DOI:** 10.1002/brb3.1303

**Published:** 2019-05-21

**Authors:** Ann Sörbo, Ingrid Eiving, Pia Löwhagen Hendén, Silvana Naredi, Johan Ljungqvist, Helena Odenstedt Hergès

**Affiliations:** ^1^ Department of Clinical Neuroscience, Institute of Neuroscience and Physiology, Sahlgrenska Academy University of Gothenburg Gothenburg Sweden; ^2^ Department of Neurology and Rehabilitation Södra Älvsborg Hospital Borås Sweden; ^3^ Deparment of Anesthesiology and Intensive Care Sahlgrenska University Hospital Gothenburg Sweden; ^4^ Department of Anesthesiology and Intensive Care, Institute of Clinical Sciences Sahlgrenska Academy, University of Gothenburg Gothenburg Sweden; ^5^ Department of Neurosurgery, Institute of Neuroscience and Physiology, Sahlgrenska Academy University of Gothenburg Gothenburg Sweden

**Keywords:** aneurysmal subarachnoid hemorrhage, mental fatigue, outcome assessment

## Abstract

**Background:**

Mental fatigue, as part of cognitive dysfunction, has been reported to be common after subarachnoid hemorrhage and it significantly affects quality of life.

**Aims of the Study:**

The aim of this study was to assess mental fatigue one year after an aneurysmal subarachnoid hemorrhage and to correlate the degree of mental fatigue to functional outcome assessed with the Extended Glasgow Outcome Scale (GOSE).

**Methods:**

One year after an aneurysmal subarachnoid hemorrhage, the GOSE was assessed and a questionnaire for self‐assessment of mental fatigue, the Mental Fatigue Scale, was distributed to all included patients. The maximum score is 42 and a score of ≥10.5 indicates mental fatigue.

**Results:**

All patients with GOSE 8, indicating full recovery, had a mental fatigue score of <10.5. A linear correlation between the GOSE and the mental fatigue score was observed (*p* < 0.0001).

**Conclusions:**

Patients with a favorable outcome and GOSE 5–7 could benefit from the assessments of mental fatigue in order to receive satisfactory rehabilitation.

## 1 INTRODUCTION

Cognitive dysfunction including mental fatigue has been reported to be common in survivors of a subarachnoid hemorrhage (SAH), even in patients with a good functional outcome as assessed by standard outcome measurements. Cognitive dysfunction can persist over a period of years and affect daily life (Al‐Khindi, Macdonald, & Schweizer, 2010; Kutlubaev, Barugh, & Mead, 2012; Macdonald & Schweizer, 2017). Mental fatigue interferes with daily life, such as the ability to return to work, general well‐being and social and recreational life (Kutlubaev et al., 2012).

The aim of this study was to assess mental fatigue 1 year after an SAH and correlate it to functional outcome as measured with the Extended Glasgow Outcome Scale (GOSE) (Wilson, Pettigrew, & Teasdale, 1998).

## 2 MATERIAL AND METHODS

The study was approved by the regional ethical review board in Gothenburg, Sweden (no. 053‐15). Patients with an aneurysmal SAH (aSAH) admitted to Sahlgrenska University Hospital, Gothenburg Sweden, were enrolled between May 2015 and October 2016. Informed consent was obtained from the patients or their legal representatives prior to inclusion. The inclusion criteria were age ≥18 and an aSAH verified by digital subtraction angiography. The exclusion criteria were a previous aSAH, stroke, or brain injury. On admission, the clinical condition was scored according to the World Federation of Neurological Surgeons (WFNS) scale (Report WFNS, 1988) and the amount of blood in the subarachnoid space was evaluated by Fisher's scale (Fisher, Kistler, & Davis, 1980).

Patients were primarily treated at the intensive care unit, in accordance with a local protocol, consistent for the most part with the American Heart Association/American Stroke Association guidelines (Connolly et al., 2012). The aneurysms were usually secured within 24 hr after admission. Nimodipine (Nimotop^®^) was administered intravenously as prophylactic treatment for vasospasm. Patients developing hydrocephalus received a ventricular catheter for cerebrospinal fluid drainage. Delayed cerebral ischemia was defined as a clinical deterioration or radiological findings according to the definition formulated by Vergouwen et al. (2010).

One year after the aSAH, the GOSE was assessed by a telephone interview and a questionnaire for the self‐assessment of mental fatigue using the Mental Fatigue Scale (MFS) was sent to the patients. The MFS scale was developed to evaluate mental fatigue in patients with neurological disorders (Johansson, Starmark, Berglund, Rödholm, & Rönnbäck, 2010).

The GOSE ranges from 1; dead to 8; full recovery. A favorable outcome was defined as GOSE 5–8 and an unfavorable outcome as GOSE 1–4 (Al‐Khindi et al., 2010; Kutlubaev et al., 2012; Macdonald & Schweizer, 2017; Wilson et al., 1998). The MFS questionnaire comprises 15 questions in different areas associated with mental fatigue; fatigue in general, lack of initiative, mental recovery, concentration difficulties, memory problems, slowness of thinking, sensitivity to stress, increased tendency to become emotional, irritability, sensitivity to light and noise, and decreased or increased duration of sleep (Johansson et al., 2010). The answers to the first 14 questions are based on what the patient has experienced during the past month. The last question, no. 15, assesses diurnal variations. The rating of each item is based on intensity, frequency, and duration. The minimum score is zero and the maximum score 42. Healthy controls report a mean score of approximately 5, while a score of ≥10.5 indicates mental fatigue (Johansson & Rönnbäck, 2014; Johansson et al., 2010).

### 2.1 Statistical analysis

Comparisons between groups were made using Fisher's exact test and correlations between the GOSE and MFS were made using Pearson's *r*. The data are presented as the mean ± *SD* or the median (range).

## 3 RESULTS

A total of 64 patients were enrolled, two were lost to follow‐up.

Basic characteristics and outcome are presented in Table 1.

**Table 1:** Basic characteristics and outcome variables

Characteristic	62 patients GOSE^a^ assessed	44 patients GOSE+MFS^b^ assessed
*Gender*		
Male/female, n *(%)*	16/46 *(26/74)*	11/33 *(25/75)*
*Age (years)*		
Median *(range)*	58 *(36–78)*	57 *(36–76)*
*Neurosurgical intervention,* n *(%)*		
Surgical clipping	16 *(26)*	8 *(18)*
Endovascular coiling	46 *(74)*	26 *(59)*
*Aneurysm location,* n *(%)*		
Anterior cerebral circulation^c^	45 *(73)*	31 *(70)*
Posterior cerebral circulation^d^	17 *(27)*	13 *(30)*
*CSF* e *drainage,* n *(%)*	32 *(52)*	19 *(43)*
*Fisher scale,* n *(%)*		
1	5 *(8)*	4 *(9)*
2	1 *(2)*	0 *(0)*
3	25 *(40)*	21 *(48)*
4	31 *(50)*	19 *(43)*
*WFNS* f * grade,* n *(%)*		
1	17 *(27)*	14 *(32)*
2	24 *(39)*	18 *(40)*
3	2 *(3)*	2 *(5)*
4	11 *(18)*	6 *(14)*
5	8 *(13)*	4 *(9)*
*Outcome*		
One‐year survival, n *(%)*	57 *(92)*	44 *(100)*
DCI^g^, n *(%)*	16 *(26)*	13 *(30)*
GOSE median *(range)*	5 *(1–8)*	6 *(4–8)*
MFS median *(range)*		13.5 *(0–31)*

a Glasgow Outcome Scale Extended.

b Mental Fatigue Scale.

c Includes anterior, middle, and anterior communicating arteries and their branches.

d Includes basilar, vertebral, and posterior communicating arteries and their branches.

e Cerebrospinal fluid.

f World Federation of Neurological Societies Grading System.

g Delayed cerebral ischemia defined as cerebral infarction and/or clinical deterioration attributable to DCI (Vergouwen et al., 2010).

**Figure 1:**
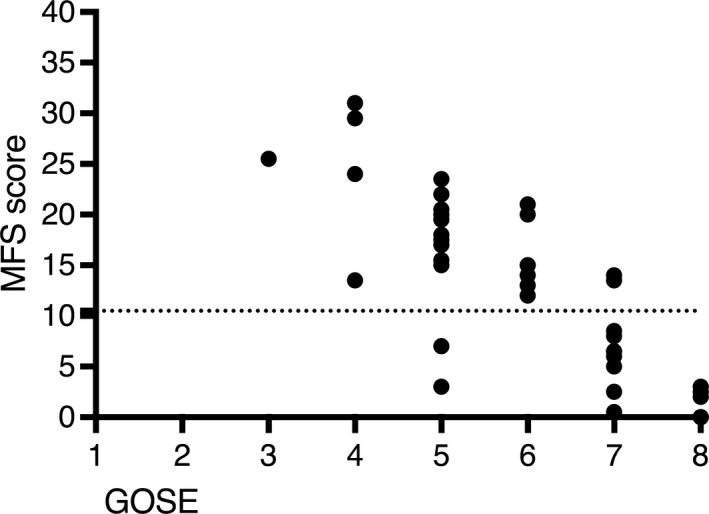
Correlation between Glasgow outcome scale extended and Mental Fatigue Scale score. Functional outcome measured with the extended Glasgow outcome scale (GOSE) in relation to the mental fatigue scale (MSF) score. GOSE 5–8 indicate a favorable outcome. The dotted line denotes an MSF score of 10.5, a score of ≥10.5 indicates mental fatigue

The median GOSE in the 62 included patients was 5 (1–8). Forty‐four of 62 (71%) had a favorable outcome (GOSE 5–8) and 18/62 (29%) had an unfavorable outcome (GOSE 1–4). No patient was in a vegetative state (GOSE 2). Five patients died within 1 year, eight were unable to complete the MFS questionnaire (GOSE 3 *n* = 6, GOSE 4 *n* = 2) and five did not return the MFS questionnaire (GOSE 5 *n* = 2, GOSE 6 *n* = 1, GOSE 7 *n* = 2), leaving 44 patients with both GOSE and MFS score assessments. The median GOSE in the 13 patients who did not complete or return the MFS questionnaire was 4 (3–7) and 39% had a favorable outcome. Among the 44/62 (69%) patients completing the 1‐year follow‐up with both a GOSE and an MFS assessment, the median GOSE was 6 (3–8) and 39/44 (89%) had a favorable outcome. The median MFS score was 13.5 (0–31) and 25/44 (57%) had an MFS score of ≥10.5, consistent with mental fatigue. No patient with GOSE 8, indicating full recovery, had an MFS score of ≥10.5. There was a correlation between GOSE and MFS scores (*p* < 0.0001) (Figure 1). Patients with GOSE 7–8 had MFS scores of ≥10.5 significantly less frequently than patients with GOSE <7 (*p* < 0.0001).

## 4 DISCUSSION

Improved treatment has reduced mortality after aSAH over the past three decades and the percentage of survivors who, according to standard follow‐up scales, attain an independent life is estimated to be around 55% (Macdonald & Schweizer, 2017).

However, even in patients scored as independent survivors after aSAH, cognitive dysfunction including mental fatigue is common (Al‐Khindi et al., 2010; Kutlubaev et al., 2012). It is important to learn more about the cognitive and functional deficits that occur after aSAH in order to offer the most satisfactory rehabilitation. In a Swedish study, cognitive symptoms including mental fatigue, impaired memory, and concentration problems were still present 5 years after aSAH (Persson, Carlsson, & Sunnerhagen, 2018). Mental fatigue has not been clearly defined until recently and a more specific assessment of mental fatigue after aSAH has not been routinely performed (Johansson & Rönnbäck, 2014).

aSAH occurs at a relatively young age and it is important to detect mental fatigue so that a patient may be able to return to work and other social activities. The rehabilitation needs to include education on how to use coping strategies, as well as offering and evaluating pharmacological treatment in order to perceive the highest possible quality of life (Johansson, Wentzel, Andréll, Rönnbäck, & Mannheimer, 2017). In this study, we found a correlation between the GOSE and the MFS scale. Only patients who scored GOSE 8 did not experience signs of mental fatigue which is consistent with the definition of GOSE 8 (no deficits) (Wilson et al., 1998), but the results indicate that patients with a favorable functional outcome of GOSE 5–7 could benefit from being assessed for the detection of mental fatigue. Eight patients with GOSE 3 and 4 were unable to answer the MFS questionnaire, meaning that the mental fatigue scale used in this study, even though some GOSE 3 and 4 patients completed the MSF questionnaire, is most suited to patients with GOSE ≥5. However, the neurological deficits associated with an unfavorable outcome probably overshadow the problems associated with mental fatigue, and it might therefore not be so relevant to assess mental fatigue in such detail for patients with GOSE <5.

A limitation of this study is that GOSE was scored from a telephone interview and in patients with a low functional outcome, a next of kin provided the answers that formed the basis for the GOSE assessment. A possible bias due to an over or underestimation by the relative could not be ruled out. However, as we used a structured form constructed for an interview, we suggest that GOSE assessment was accurately performed.

Another limitation is that GOSE has ceiling affects by being insensitive to subtle deficits of function. This scale is however widely used and was the instrument with greatest responsiveness and the lowest ceiling effect in a major trauma population with and without significant head injuries (Williamson et al., 2011).

In summary the Mental Fatigue Scale score after an aSAH was significantly correlated to the GOSE. As suspected no patient with GOSE 8 experienced mental fatigue. Patients with GOSE 5–7 could however benefit from a more standardized assessment of mental fatigue. We should be aware of fatigue as one of the factors of impaired function in patients who achieved good recovery, moderate disability and are independent in daily living so that satisfactory rehabilitation for the improvement of long‐term outcome can be offered.

## CONFLICT OF INTEREST

None of the authors have any conflict of interest.

## DATA AVAILABILITY STATEMENT

The data that support the findings of this study are available from the corresponding author upon request.
